# Spontaneous Knee Hemarthrosis Due to Hypofibrinogenemia Following Tigecycline Treatment for Periprosthetic Joint Infection

**DOI:** 10.7759/cureus.5883

**Published:** 2019-10-10

**Authors:** Theodore Balfousias, Alexandros P Apostolopoulos, Stavros Angelis, Spyridon Maris, Athanasios Papanikolaou

**Affiliations:** 1 Orthopaedics, General Hospital Hellenic Red Cross Korgialenio Benakio, Athens, GRC; 2 Orthopaedics, East Surrey Hospital, Surrey and Sussex Healthcare National Health Service Trust, Redhill, GBR; 3 Orhopaedics, General Hospital Hellenic Red Cross Korgialenio Benakio, Athens, GRC

**Keywords:** tigecycline, periprosthetic infection, hypofibrinogenemia, hemarthrosis, adverse effect

## Abstract

Tigecycline, a recently approved antibiotic, has a broad spectrum of antimicrobial activity. Its unique structure and properties make tigecycline a valuable option for the treatment of infections caused by many multidrug-resistant organisms. We present a case of an 82-year-old patient who developed a significant decrease of fibrinogen levels after the addition of tigecycline to his antibiotic regimen. The patient was treated for a periprosthetic knee joint infection caused by a multidrug-resistant extended-spectrum beta-lactamase-producing Escherichia coli. The reduction of fibrinogen levels, in this case, prompted severe spontaneous hemarthrosis. Tigecycline treatment was discontinued and coagulation disorders were normalized within the next few days. After several days, the joint had to be surgically debrided. Hypofibrinogenemia is a very scarcely reported side effect of tigecycline that can cause spontaneous hemarthrosis.

## Introduction

Tigecycline is the first antibiotic in the class of glycylcyclines, a class that has structural similarities with tetracycline. Tigecycline, which was recently approved by the Food and Drug Administration (FDA) in 2005 and in Europe in 2006, has a broad spectrum of antimicrobial activity. Modifications have been made to the tetracycline structure. These modifications make tigecycline active against tetracycline-resistant organisms. In addition to tigecycline’s activity against most gram-positive, gram-negative, and anaerobic bacteria, it is considered effective against many multi-drug resistant organisms (MDROs). Methicillin-resistant* Staphylococcus aureus (MRSA), *vancomycin-resistant Enterococci (VRE), Acinetobacter Baumannii, and extended-spectrum beta-lactamase (ESBL)-producing *Enterobacteriaceae* are some of the multidrug-resistant bacteria susceptible to tigecycline [1].

Various adverse events, related to the administration of tigecycline, have been reported in the literature. Gastrointestinal symptoms, including nausea and vomiting, are the most common [2-3]. Acute pancreatitis, liver dysfunction, as well as elevated levels of aminotransferases, bilirubin, and alkaline phosphatase, have also been reported. Coagulation disorders are very uncommon following treatment with tigecycline. Hypofibrinogenemia, caused by tigecycline, is a scarcely reported side effect. We present a patient, who was treated with tigecycline for periprosthetic infection of the knee that developed hypofibrinogenemia and led to severe spontaneous knee hemarthrosis.

## Case presentation

An 82-year-old male patient was referred to our emergency department due to a neglected periprosthetic knee joint infection of his left knee. He had undergone a primary total knee arthroplasty 11 years ago in another institution because of painful osteoarthritis. Failure of the primary arthroplasty led to revision surgery two years later, at the same institution. The patient reported prolonged mild edema and moderate pain subsequent to the revision surgery and deterioration of symptoms during the past two months.

**Figure 1:**
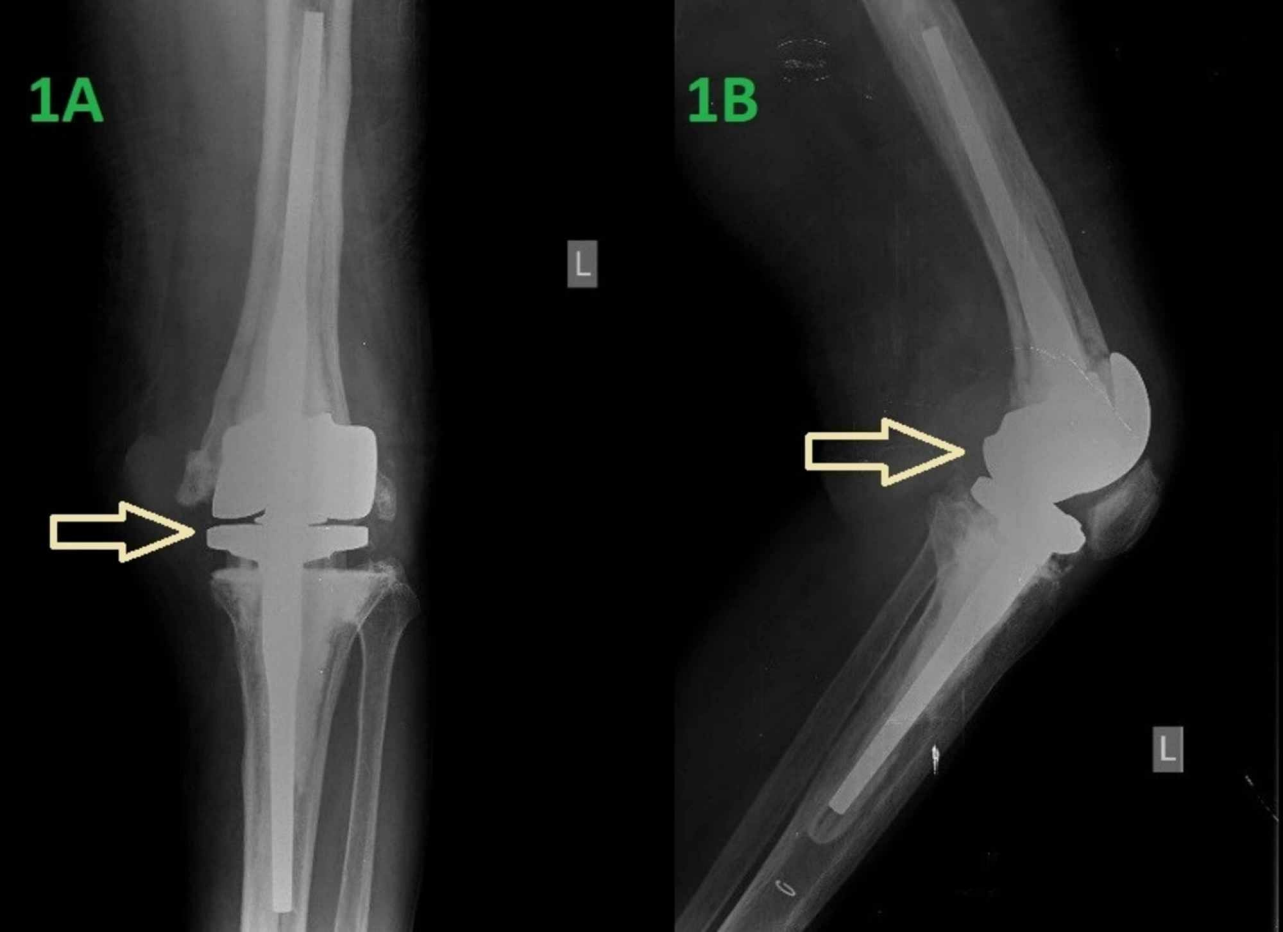
Initial X-rays of the patient's knee revealing the revision prosthesis (A): anteroposterior view (B): lateral view

On admission, the patient reported severe pain in the left knee. This was the reason for the limitation of his daily activities. The patient was afebrile and no injury in the recent past was referred. Τhe knee joint was excessively swollen, with prominent erythema and warmth. Moreover, imaging by X-ray revealed a constrained revision prosthesis and no signs of fracture (Figure 1). The range of motion was affected, with a major deficit in flexion and a 10 degrees deficit in extension.

An ultrasound of the knee joint was performed in the emergency department and revealed massive fluid accumulation in the joint. Blood samples revealed abnormal inflammatory markers. The C-reactive protein (CRP) was 20.3 mg/L and the erythrocyte sedimentation rate (ESR) was 67 mm/hr, but no leukocytosis was observed. Blood cultures were also obtained. The rest of the standard laboratory evaluation was normal.

As far as medical history is concerned, the patient reported myelodysplastic syndrome (MDS) and benign prostatic hyperplasia (BPH) under treatment. MDS could possibly be one of the reasons why leucocytosis was absent, even though, in many cases of chronic musculoskeletal infections, the white blood cell count is normal. The platelet count was normal (PLT: 262 X10^3^/μL) and the red blood cell count (RBC) was 4.25 x10^6^/μL with haemoglobin 11.1 g/dL. No known allergic reactions were reported.

Surgical debridement of the joint was decided, and it took place in the operation room on the same day. During the procedure, pus was removed from the joint, several cultures were taken, and the joint was excessively debrided. After the operation, broad-spectrum empiric intravenous antibiotic therapy was initiated, consisting of piperacillin and tazobactam (4 g + 0.5 g) every six hours and vancomycin 1 g every 12 hours. Prophylactic dosage of low molecular weight heparin (LMWH) was also administered subcutaneously (enoxaparin sodium 4,000 IU once daily).

The cultures from the joint fluid and tissues revealed a multidrug-resistant extended-spectrum beta-lactamase (ESBL)-producing *Escherichia coli*. ESBLs are enzymes that hydrolyze most beta-lactamase antibiotics, including penicillins, cephalosporins, and monobactams. On the other hand, blood cultures turned negative. Seven days after surgical debridement, the antibiotic regimen changed, based on the antibiogram. In collaboration with the department of infectious diseases of the hospital, therapy with intravenous meropenem and high dosage of tigecycline was initiated. The patient received a loading dose of 200 mg tigecycline followed by 100 mg every 12 hours in combination with 2 g meropenem every eight hours.

Six days after the initiation of the therapy with tigecycline, the patient reported complaints of abdominal pain and nausea. No vomiting was referred. Liver function tests, as well as heart function tests and electrocardiogram (ECG), were normal. The abdominal ultrasonography did not reveal any abnormal findings besides the size of the spleen, which was at the upper normal limits. The symptoms subsided with the administration of proton-pump inhibitors (PPIs).

**Figure 2:**
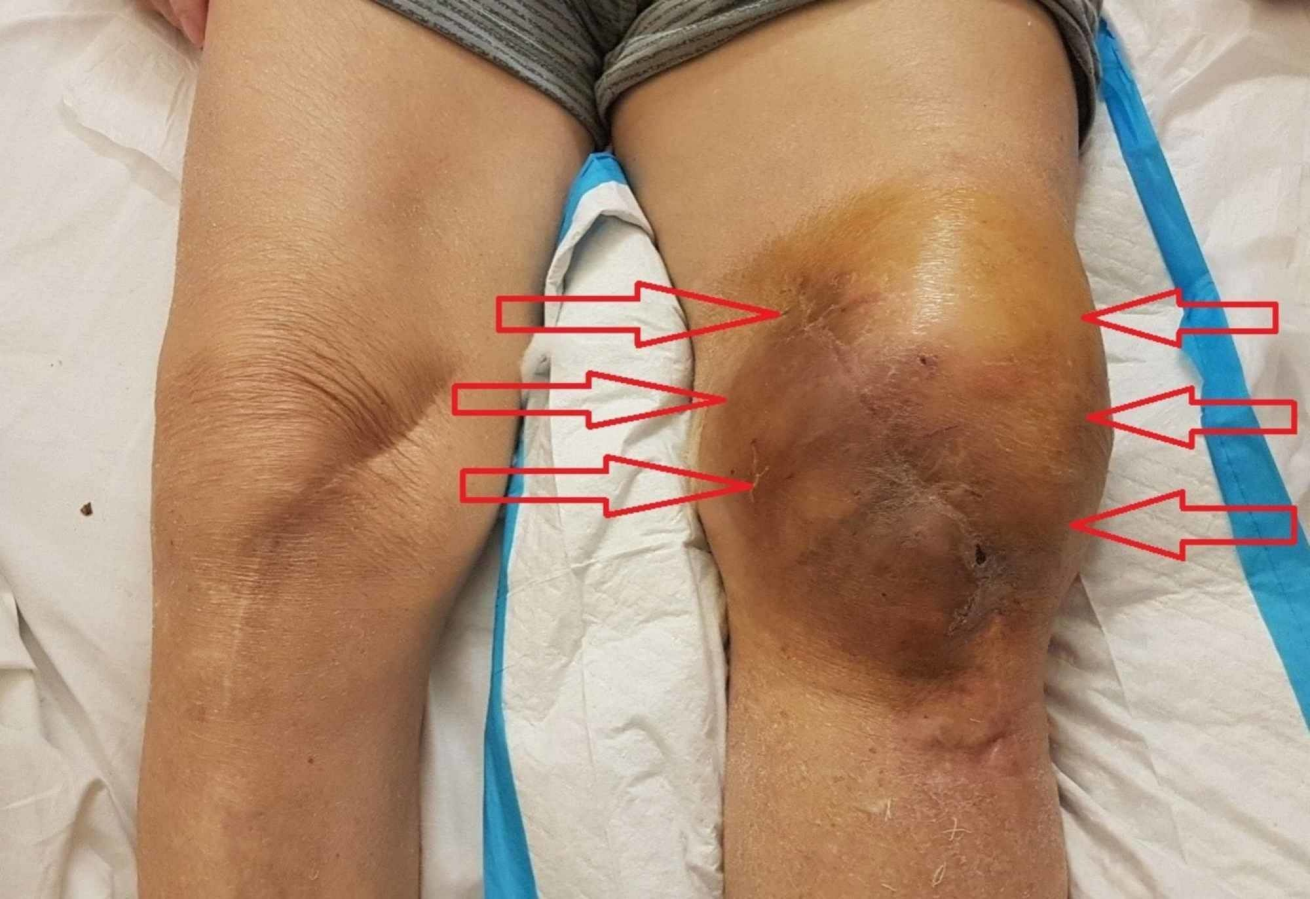
Severe hemarthrosis of the left knee after 16 days of tigecycline administration

Fourteen days after initial tigecycline dosage administration, spontaneous swelling appeared on the left knee. Although systemic inflammatory markers and the patient's general condition were improving, the swelling of the joint progressively worsened within the next two days, despite ice therapy (Figure 2). Simultaneously, a prolongation of the activated partial thromboplastin time (aPTT) and the international normalized ratio (INR) was noted, along with a vast decrease of fibrinogen (FIB) levels. On the other hand, the platelet count slightly decreased but remained in the normal range, over 150 X10^3^/μL. Liver function blood tests were also normal.

Joint aspiration was performed and 120 mL of blood was drained. The aspirate was sent for culture and sensitivity. Administration of low molecular weight heparin (LMWH) was interrupted. Fluid cultures were negative. Lack of platelet consumption, the good general condition of the patient, and an improvement in inflammation markers led us to rule out other causes of hypofibrinogenemia such as disseminated intravascular coagulation (DIC) and sepsis.

Hypofibrinogenemia worsened 18 days after the initiation of tigecycline treatment, with fibrinogen (FIB) reaching 158 mg/dL, aPTT 54.3 s, INR 1.78, CRP 11.8 mg/L, platelet count 181 X10^3^/μL, white blood cell count (WBC) 4.3 x10^3^/μL, haemoglobin 9.1 g/dL, and normal liver function tests. The hemarthrosis of the knee worsened, and the patient complained of severe pain of the joint. It was suspected that tigecycline was the causative factor of the hypofibrinogenemia that led to severe hemarthrosis. The antibiotic was discontinued, and the new antibiotic regimen consisted solely of intravenous administration of meropenem. During the next three days, eight fresh frozen plasma units were administered, along with vitamin K and 1 g of intravenous tranexamic acid. Following the discontinuation of tigecycline, the coagulation disorders became normal through the next six days. The fibrinogen levels reversed to the normal range (Table 1).

**Table 1:** Coagulation parameters and inflammatory markers of the patient on admission, during tigecycline therapy and after drug discontinuation INR: International Normalized Ratio; PT: Prothrombin Time; aPTT: Activated Partial Thromboplastin Time; FIB: Fibrinogen; PLT: Platelet Count; CRP: C-Reactive Protein; ESR: Erythrocyte Sedimentation Rate

	Day of admission	1^st^ day in tigecycline treatment	14^th^ day in tigecycline treatment	18^th^(final) day of tigecycline	3 days after tigecycline discontinuation	6 days after tigecycline discontinuation
INR	1.12	1.15	1.64	1.78	1.30	1.14
PT (s)	15.2	14.9	20.8	22.1	17.1	15.1
aPTT (s)	40.0	40.0	48.5	54.3	43.2	39.1
FIB (mg/dL)	398	393	202	158	364	396
PLT (X10^3^/μL)	262	197	226	181	146	149
CRP (mg/L)	20.3	16.1	12.4	11.8	14.2	8.6
ESR (mm/hr)	67	48	36	28	31	18

**Figure 3:**
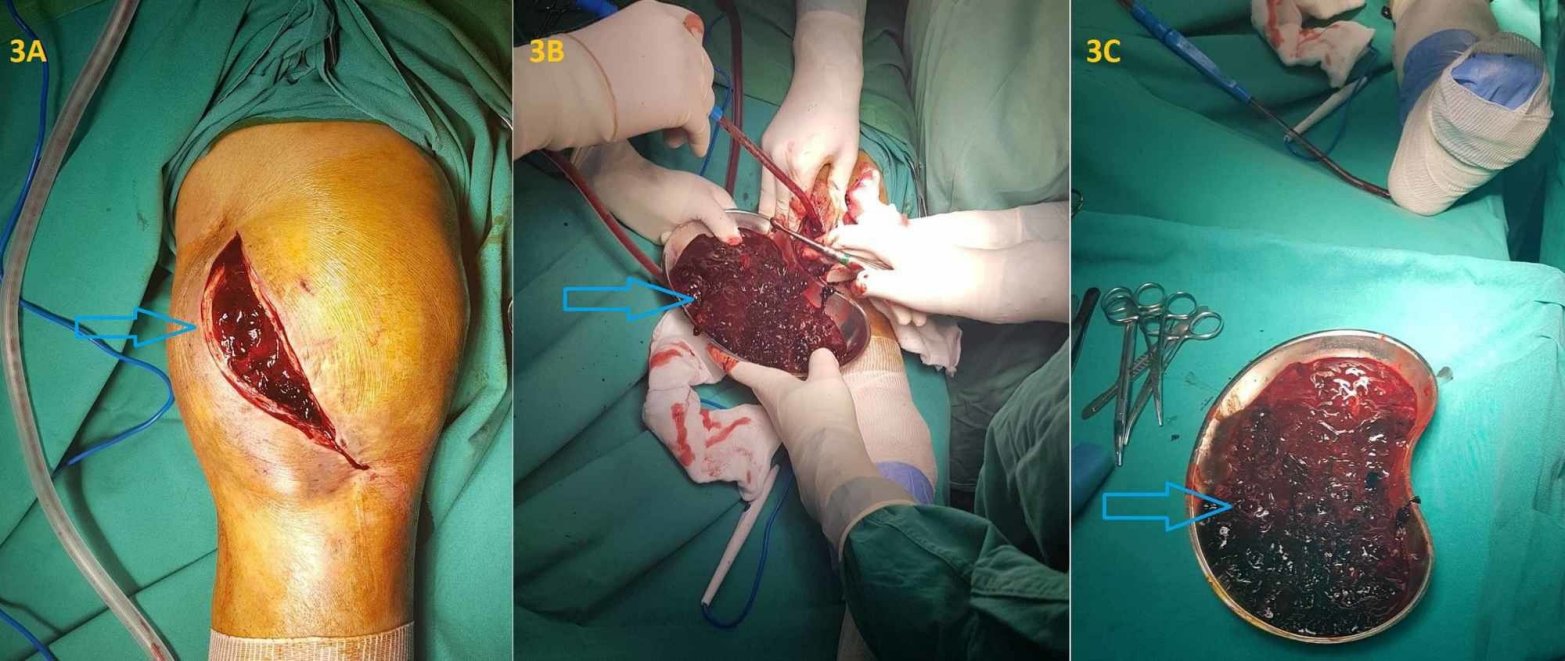
Surgical debridement of the knee 10 days after the discontinuation of tigecycline, which showed excessive clotted blood accumulation

This improvement was consistent with our initial hypothesis that tigecycline caused the coagulation disfunction. Despite the gradual improvement in hypofibrinogenemia, the severe hemarthrosis had to be surgically treated. The joint had to be debrided in the operation room 10 days after the discontinuation of tigecycline, as blood accumulation could not be aspirated because of blood clots. About 500 mL of clotted blood was obtained after the surgical debridement and no obvious site of hemorrhage was revealed (Figure 3). No recurrence of hemarthrosis during hospitalization was observed.

## Discussion

Tigecycline is a broad-spectrum, tetracycline analog antibiotic. It blocks protein synthesis, as it binds to the 30S subunit of the ribosome, inhibiting the proliferation of multiple bacteria. It is an active antibiotic against many MDROs, with the exception of *Proteus, Providencia, *and *Pseudomonas aeruginosa *[4]. Its use has been approved by the Food and Drugs Administration (FDA) and the European Medicines Agency (EMA) for complicated intra-abdominal infections, community-acquired pneumonia by sensitive bacteria, and complicated skin and soft tissue infections. However, tigecycline has recently been used in the treatment of periprosthetic joint infections [5-6]. Treating periprosthetic joint infections is a challenge because of the presence of a microbial biofilm. Treatment of these kinds of infections requires the surgical removal of all implants and intravenous antibiotic administration.

In our case, the patient refused to undergo new revision surgery and to replace the prosthesis. Biofilms are complex structures that act as protection of the bacteria from the host defenses and from the antibiotics that are administered. In a recent in vitro study, tigecycline, along with rifampicin, showed superior activity against a *Staphylococcus epidermidis* biofilm in comparison with ciprofloxacin, vancomycin, cloxacillin, and daptomycin [7]. Additionally, animal studies show that tigecycline is active against foreign body infections, as monotherapy or in combination with other antimicrobial agents [8-9]. Although there is limited experience in the treatment of periprosthetic joint infection because of the biofilm and the MDRO, the use of high dosage tigecycline administration was chosen to maximize the effectiveness of the drug [6,10].

The poor bioavailability of tigecycline necessitates its intravenous administration. Tigecycline has a relatively long elimination half-life (t 1/2) of 42 hours and reaches steady serum concentration levels after seven days of therapy [3]. It is metabolized mainly by the liver, hence, a reduction in the dosage is recommended in patients with Child-Pugh class C cirrhosis [4]. The recommended dosage is a loading 100 mg (IV, intravenous) the first day of the therapy, followed by 50 mg (IV) every 12 hours the next days. However, there is a debate about the optimal dosage, as there are several publications reporting the better efficiency of tigecycline in high dose administration [10].

Symptoms of the gastrointestinal (GI) tract are the most common side effects of tigecycline. In the clinical trials performed, coagulation disorders, such as the prolongation of INR, aPTT, and prothrombin time (PT), were very infrequently (<2%) reported in patients receiving tigecycline treatment though a reduction in fibrinogen levels was not described [11]. After reviewing the literature for cases of hypofibrinogenemia following the use of tigecycline, six case reports were found published in PubMed after the widespread use of tigecycline worldwide [12-17]. In all cases, hypofibrinogenemia was reversible and the coagulation disorders resolved within days after stopping the administration of the antibiotic. Pieringer et al. were the first to report, in 2009, a case of hypofibrinogenemia that developed after tigecycline was added to the antibiotic regimen of a patient, with no underlying hepatic disease, who was treated for peritonitis. Five days after the discontinuation of tigecycline, the fibrinogen levels returned to normal values [12].

Three small-scale studies are known to assess the effect of tigecycline treatment on fibrinogen levels. Routsi et al. studied the coagulation parameters of 45 patients intensive care unit (ICU) patients and showed that high doses of tigecycline led to a gradual reduction in fibrinogen levels and the prolongation of INR and aPTT, 14 days after the initiation of the treatment. The coagulation parameters returned to normal after the discontinuation of the treatment, although the coagulation parameters could not be assessed in 15 patients, either because of death, non-drug related, or because of discharge from the intensive care unit (ICU) [18]. In another study, Zhang et al. evaluated 20 patients with severe infections, who were treated with tigecycline. Hypofibrinogenemia was described in 80% of the patients, which was proportional to the dose administered. There was no significant difference between age groups. The reduction of FIB levels was reversed after the cessation of treatment [19]. Leng et al. retrospectively studied 50 patients treated with tigecycline and monitored their coagulation parameters. Forty-six of the patients had a reduction of fibrinogen levels and the reduction was more profound in 25 of them. They also found that the alterations in FIB, aPTT, and PT started to appear four days after the initial dosage, and they worsened over the following days of treatment. In this study, the changes in the coagulation parameters gradually reversed four days after the discontinuation of treatment [20].

Fibrinogen is a soluble protein, with a half-life of four days; it is produced in hepatocytes. It has a major role in coagulation, as it is converted into insoluble fibrin. It is also one of the acute phase proteins and elevated levels of fibrinogen are expected in systematic inflammation and malignancy. There are rare inherited diseases such as afibrinogenemia, hypofibrinogenemia, and dysfibrinogenemia. Moreover, a reduction in fibrinogen levels can be seen in hepatic diseases and malnutrition. Disseminated intravascular coagulation (DIC), transfusions of large amounts of blood, and the administration of drugs (valproic acid, synthetic adrenocorticotropic hormone (ACTH), prednisolone, L-asparaginase, allopurinol, and, recently, tigecycline) can also lead to a reduction in the levels of fibrinogen [14]. The exact mechanism by which tigecycline causes hypofibrinogenemia is ambiguous. It is a member of glycylcyclines with structural similarities to tetracyclines. It can impair the patient’s coagulation either by affecting the vitamin-K-producing flora of the gastrointestinal (GI) tract or by directly provoking alterations on the coagulation cascade [18]. In addition, a possible effect on the hepatic function that leads to impaired fibrinogen levels has also been hypothesized [14].

In our case, the patient had no history of coagulation disorder or hepatic disease. As the inflammatory markers were improving during the treatment and there was no platelet consumption noted, excluding other causes of reduction of fibrinogen levels, our hypothesis was that hypofibrinogenemia was the result of tigecycline administration. The coagulation parameters, including fibrinogen (FIB) levels, became normal six days after the discontinuation of the antibiotic, confirming our assumption. To our knowledge, this is the first case reported that tigecycline-induced coagulopathy led to an acute, painful hemarthrosis that had to be encountered in the operation room.

## Conclusions

Hypofibrinogenemia, as an adverse event of tigecycline, should be considered in the event of a spontaneous hematoma. In cases treated with antibiotic regimens, which include tigecycline, we suggest thorough monitoring of the coagulation parameters (aPTT, PT, INR, and fibrinogen). The discontinuation of tigecycline is advised for patients that develop hypofibrinogenemia, especially if there are no other apparent causes present.
